# Fetal lung interstitial tumor (FLIT): a case report and review of prenatal imaging features

**DOI:** 10.1007/s00247-026-06732-w

**Published:** 2026-07-24

**Authors:** An T. Hoang, Zeynep Ruhioglu, David Carpentieri, Luis F. Goncalves

**Affiliations:** 1 Creighton University, 3100 N Central Ave, Phoenix, AZ 85012 United States; 2 Department of Radiology, Phoenix Children’s Hospital, Phoenix, United States; 3 Department of Pathology, Phoenix Children’s Hospital, Phoenix, United States; 4 Department of Radiology, Mayo Clinic, Scottsdale, United States

**Keywords:** Fetal lung interstitial tumor, Fetal lung mass, Hydrops

## Abstract

Fetal lung interstitial tumor (FLIT) is a rare benign mesenchymal lung tumor that is often mistaken for more common congenital lung lesions such as congenital pulmonary airway malformation (CPAM). Prenatal detection is exceedingly uncommon. Here, we report a case of prenatally detected FLIT presenting in the third trimester with rapid interval growth, mediastinal shift, and fetal hydrops, raising concern for a malignancy, including pleuropulmonary blastoma. Prenatal ultrasonography and fetal magnetic resonance imaging (MRI) demonstrated a rapidly enlarging right lung mass first identified late in gestation with associated findings of pleural effusion, ascites, and diffuse skin edema consistent with hydrops. Progressive mass effect prompted expedited delivery. Postnatal imaging confirmed a large pulmonary lesion, and the neonate developed significant respiratory distress requiring lobectomy. Histopathologic evaluation revealed an immature mesenchymal proliferation diagnostic of FLIT. This case highlights the imaging overlap between FLIT, CPAM, and pleuropulmonary blastoma and underscores the importance of considering FLIT in the differential diagnosis of late-presenting fetal lung masses. Although imaging is essential for detection and prenatal planning, definitive diagnosis requires histopathologic confirmation, and complete surgical resection yields an excellent prognosis.

**Graphical Abstract:**

**Figure:**
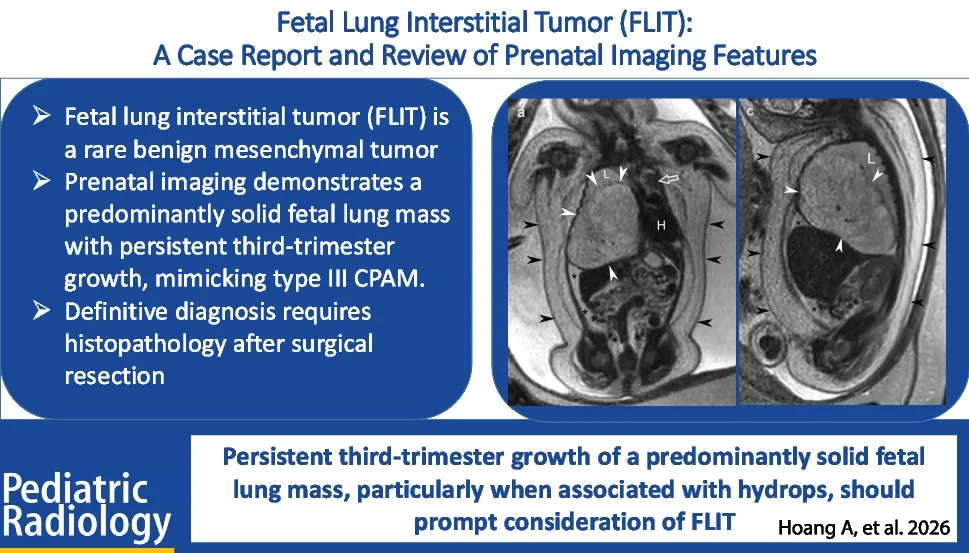

## Introduction

Fetal lung interstitial tumor (FLIT) is a rare, benign mesenchymal lung tumor that presents in the perinatal period, typically detected either antenatally or within the first 3 months of life. FLIT is characterized by a well-circumscribed, solid, or mixed solid-cystic mass that histologically resembles fetal lung tissue at the canalicular stage (20–24 weeks of gestation) [1, 2]. Histologically, it consists of immature mesenchymal cells expanding the interstitium with irregular airspace-like structures but lacking features of more aggressive neoplasms, such as cytologic atypia, increased mitotic activity, or rhabdomyoblastic or other sarcomatous differentiation [2–4].

Fetal lung interstitial tumor (FLIT) is an exceptionally rare diagnosis in the prenatal setting and is considered only after more common congenital lung lesions have been excluded. The majority of prenatally detected lung masses are congenital pulmonary airway malformations (CPAMs), bronchopulmonary sequestrations, hybrid lesions, congenital lobar overinflation (CLO), or bronchial atresia, whereas primary congenital neoplastic lesions, including FLIT and pleuropulmonary blastoma (PPB), are rare. CPAMs are typically identified during routine second-trimester ultrasound and may demonstrate significant growth, exerting mass effect on the heart and mediastinum. However, growth typically stabilizes around 26–28 weeks of gestation and may regress later in gestation. In a minority of cases, continued lesion expansion can cause compression of adjacent structures, including the inferior vena cava and heart, impairing right atrial venous return and potentially resulting in fetal heart failure, hydrops, and intrauterine demise if left untreated [5–7].

When a congenital lung mass resembling a large CPAM presents for the first time during the third trimester of pregnancy, the possibility of a primary lung tumor should be strongly considered the main differential diagnosis. These include PPB, FLIT, congenital peribronchial myofibroblastic tumor (CPMT), inflammatory myofibroblastic tumor (IMT), and congenital fibrosarcoma [5].

The prenatal natural history of FLIT is poorly understood. In a recent review, a prenatal diagnosis occurred in only three of 22 cases, with the earliest prenatal diagnosis at 26 weeks [8–10]. Most cases present at birth with respiratory distress, mediastinal shift, or cardiac dysfunction, in contrast to CPAMs, which are often clinically silent [8].

Herein we report an additional case of FLIT manifesting as a large lung mass with associated fetal hydrops identified in the third trimester.

## Case report

This was a 44-year-old G6P5 woman who presented at 27 weeks’ gestation with abdominal pain. Her medical history was notable for polyhydramnios (amniotic fluid index=27 cm), obesity (BMI=36 kg/m^2^), prediabetes, and recent COVID-19 infection. The prenatal screening including a cell-free DNA testing (Panorama) was unremarkable. Laboratory findings were significant for an abnormal thyroid-stimulating hormone, elevated hemoglobin A1C, and low vitamin D levels. At 30 weeks of gestation, a lung lesion suspected to represent a congenital pulmonary airway malformation (CPAM) was noted on ultrasound. The follow-up ultrasound at 32 weeks showed no evidence of hydrops. Notably, the lesion had not been visualized on the second-trimester anatomic survey.

**Fig. 1:**
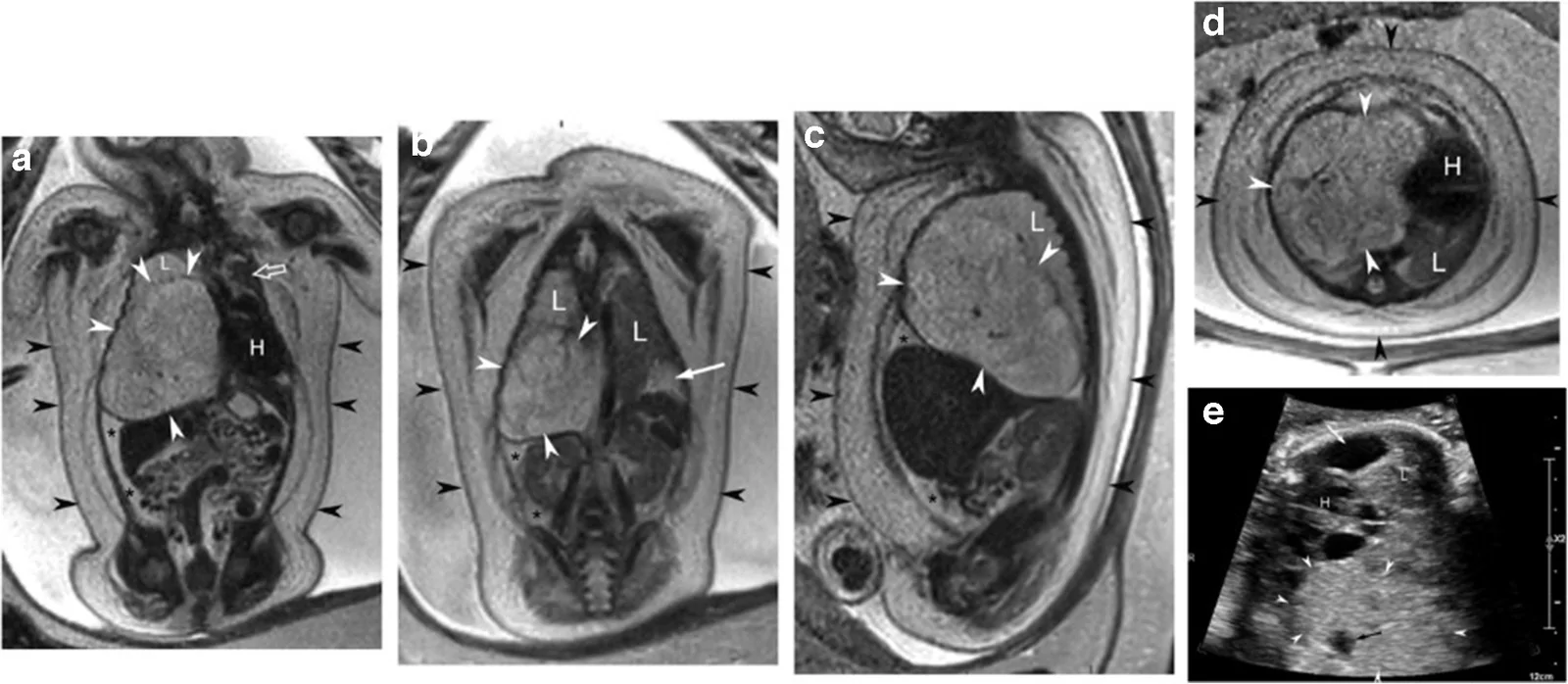
Fetal MRI at 33 weeks and 6 days demonstrates a large heterogeneous T2-hyperintense right lung mass (*white arrowheads*) with significant mediastinal shift to the left and extreme levoposition of the fetal heart (*H*) and thymus (and thymus (*open arrow*, **a**)). Single-shot fast spin echo (SSFSE) sequences are shown in coronal (**a** and **b**; **b** is posterior to **a**), sagittal (**c**), and axial (**d**) planes. Compressed lung parenchyma (*L*) is visible in (**a**), (**b**), (**c**), and (**d**). Also notable is massive body wall edema (*black arrowheads*), ascites (*asterisk*), and a left-sided pleural effusion (*white arrow*, **b**). Axial sonographic image obtained during fetal echocardiography (**e**) demonstrates a predominantly hyperechoic right thoracic mass (*white arrowheads*) containing at least scattered anechoic cysts (representative example indicated by the *black arrow*). The white arrow indicates the left pleural effusion, *H* denotes the heart, and *L* denotes compressed left lung parenchyma

Magnetic resonance imaging (MRI) performed at 33 weeks and 6 days revealed a predominantly solid T2-hyperintense lesion with a few scattered macrocystic components occupying much of the right lung, compressing the right upper and lower lobes, and displacing the mediastinum leftward with associated levoposition of the heart (Fig. 1). The lesion measured 8.3×7.3×6.0 cm, corresponding to an estimated volume of 214.9 cm^3^ and a calculated CPAM volume ratio (CVR) of 6.87 cm^2^. Compared with the reported outside ultrasound measurements, the lesion demonstrated progressive enlargement from 5.1×4.5×5.3 cm (CVR, 3.06 cm^2^) at 30 weeks’ gestation to 7.4×5.4×5.3 cm (CVR, 3.51 cm^2^) at 32 weeks’ gestation, with no evidence of hydrops at that time. Associated MRI findings included a right pleural effusion, moderate ascites, and diffuse skin edema, consistent with fetal hydrops. While CPAM remained in the differential diagnosis, the lesion’s predominantly solid appearance on ultrasound and continued growth into the third trimester, despite the typical stabilization or regression of CPAM later in gestation, raised concern for a primary pulmonary neoplasm. In retrospect, these imaging features favored FLIT; however, given its markedly different prognosis and the rare prenatal reports of mixed solid-cystic or solid PPB, it remained an important consideration [5]. The subsequent development of hydrops further supported consideration of these alternative diagnoses, although hydrops may also occur with large CPAMs.

Fetal echocardiography confirmed hydrops with pleural effusion and ascites, although cardiac anatomy was otherwise normal. Due to the rapid development of fetal hydrops within 1 week and risk of intrauterine demise, the patient underwent an emergency Cesarean delivery at 33 weeks and 6 days at an outside hospital.

**Fig. 2:**
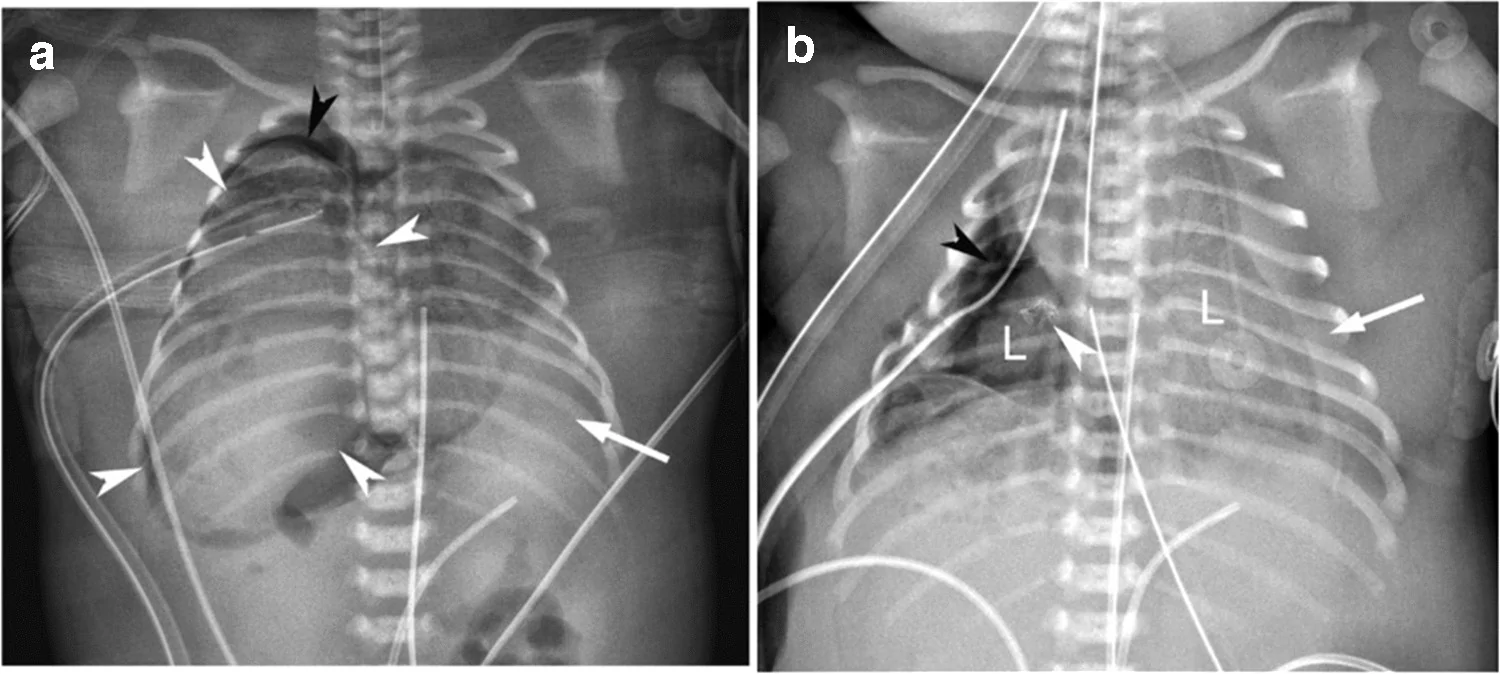
Chest radiographs obtained on day of life (DOL) 0. **a** Preoperative image demonstrates a large right-sided intrathoracic mass (*white arrowhead*), consistent with the prenatal MRI findings. The mass is associated with leftward mediastinal shift and silhouettes the cardiac border and contralateral lung. Note the right pleural air (*black arrowhead*) and left pleural effusion (*white arrow*). **b** Immediate postoperative image following resection. The white *arrowhead* indicates a surgical suture. Residual bilateral lung parenchyma (*L*) is now visible. Right pleural air (*black arrowhead*) and a left pleural effusion (*white arrow*) are also present

**Fig. 3:**
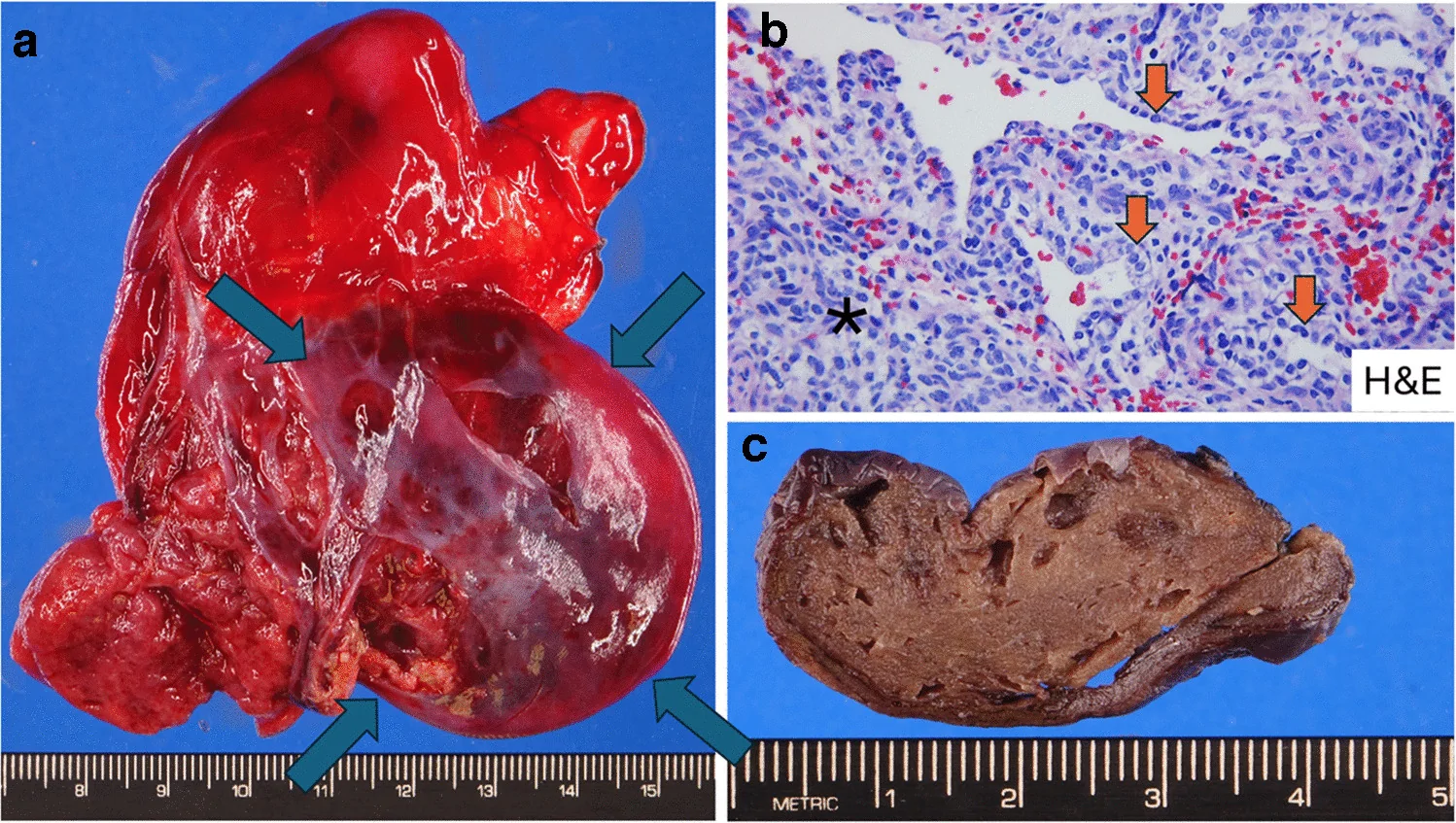
Gross and histologic features of the lesion. The lesion (*blue arrows* in **a**) involved the right upper and middle lobes and measured approximately 5-cm in the greatest dimension. The formalin fixed tissue cut surfaces (**c**) were mostly solid with few cavitary spaces. The ×20 H&E histologic section (**b**) showed a population of monotonous primitive mesenchymal cells with clear cytoplasm and round to ovoid central nuclei expanding the pulmonary interstitium (*asterisk*). Some of the airspaces were lined by a single layer of low cuboidal pulmonary epithelium (*orange arrows*). No significant cytologic atypia or mitotic activity was seen

At birth, the neonate required chest compressions, as well as multiple doses of epinephrine. The return of spontaneous circulation was achieved at 16 min of life. A chest tube was placed with serosanguinous output, and the infant was stabilized with inhaled nitric oxide and synchronized intermittent mechanical ventilation before transport to our facility for further management. On arrival at our institution, the patient underwent emergent right thoracotomy for a right upper and middle lobectomy. Figure 2a shows chest radiographs on day of life 0 with a right-sided mass, consistent with prenatal MR imaging, and additional right pneumothorax. Intraoperatively, the mass involved the entire right middle lobe and most of the right upper lobe (Fig. 3a), resulting in marked mediastinal shift. The surgery was complicated by massive hemorrhage, necessitating aggressive fluid and blood product resuscitation. The patient was subsequently transferred to the NICU for postoperative care. Immediate postoperative radiographs (Fig. 2b) demonstrated right pleural air and left pleural effusion with visualization of the remaining lung parenchyma.

These pathological features (Fig. 3) were diagnostic of fetal lung interstitial tumor (FLIT), distinguishing the lesion from CPAM and PPB.

**Fig. 4:**
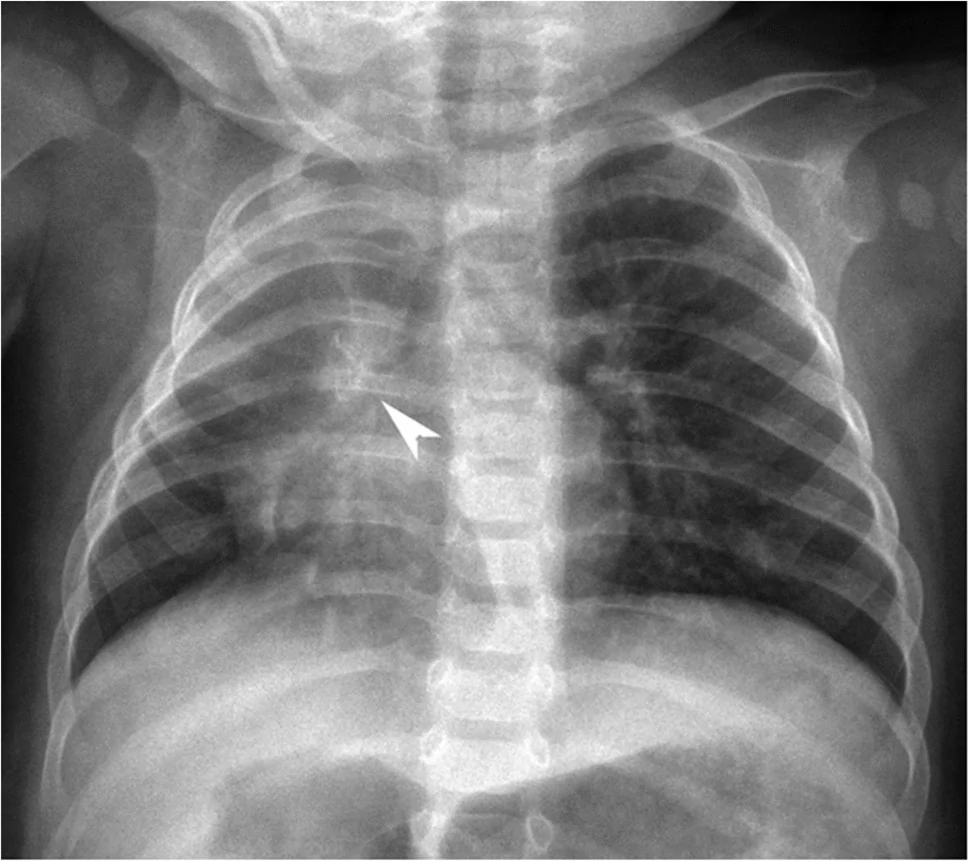
Follow-up chest radiograph obtained at 6 months of age demonstrates volume loss in the right hemithorax with mild rightward mediastinal shift. Residual right lung parenchyma is aerated. Surgical suture is visible in the right hemithorax (*white arrowhead*), marking the prior resection site. No evidence of recurrent mass, pneumothorax, or pleural effusion is seen

Postoperative computed tomography (CT) of the chest obtained at 21 days of age demonstrated expected postsurgical changes following right upper and middle lobectomies, including rightward mediastinal shift and focal subsegmental atelectatic changes in the left lower lobe, with no suspicious residual mass to suggest residual tumor. Follow-up chest imaging at 6 months of age (Fig. 4) showed expected volume loss of the right hemithorax related to surgical resection, with no evidence of tumor recurrence, pneumothorax, or pleural effusion. At the most recent pulmonology follow-up, at 1 year and 8 months of age, the patient was doing well without respiratory symptoms.

## Discussion

Fetal lung interstitial tumor (FLIT) is a rare, benign mesenchymal tumor of the lung, typically presenting in the perinatal period and often mistaken for more common congenital lung lesions such as congenital pulmonary airway malformation (CPAM) [1, 2]. To date, only a limited number of FLIT cases have been reported in the literature, underscoring the diagnostic challenge posed by this tumor. The overlap in prenatal imaging features between FLIT, CPAM, and malignant tumors such as pleuropulmonary blastoma (PPB) highlights the importance of integrating radiographic, clinical, and histopathologic data in establishing an accurate diagnosis [1–4].

Congenital pulmonary airway malformation (CPAM) is the most common prenatally detected congenital lung malformation and is often identified during the second trimester. Other common congenital lung lesions include bronchopulmonary sequestration, hybrid CPAM-sequestration lesions, congenital lobar overinflation, bronchial atresia, and bronchogenic cyst [11]. CPAM is categorized into five subtypes according to the Stocker system based on specific histopathologic features [12]. Histologically, CPAM - particularly type III lesions - may resemble FLIT because both recapitulate features of canalicular-stage fetal lung, resulting in overlapping imaging characteristics [2, 4]. Both lesions may appear as predominantly solid fetal lung masses with small cystic components, making definitive prenatal distinction difficult without histopathologic evaluation. In most cases, CPAM is detected during the second trimester and stabilizes or regresses by late gestation. By contrast, lung tumors, such as PPB and FLIT, are often undetected until the third trimester, at which point their continued growth may precipitate mass effect, mediastinal shift, or fetal hydrops, as seen in our patient [2, 10, 13, 14]. The development of hydrops is a particularly ominous finding associated with an increased risk of fetal demise, often necessitating expedited delivery and postnatal surgical intervention. In this case, the absence of a second-trimester lesion, late gestational presentation with rapid enlargement, and association with hydrops raised concern for malignant PPB, which can closely mimic FLIT radiographically but carry a markedly worse prognosis.

PPB often presents at 31–35 weeks’ gestation as a cystic lesion, closely resembling CPAM or even FLIT on imaging [8, 10]. However, unlike FLIT, PPB carries a poor prognosis and requires prompt staging, surgical resection, and chemotherapy. Thus, while imaging may suggest the presence of a congenital lung mass, histopathological evaluation is essential to accurately distinguish benign lesions like FLIT from malignant counterparts such as PPB.

**Fig. 5:**
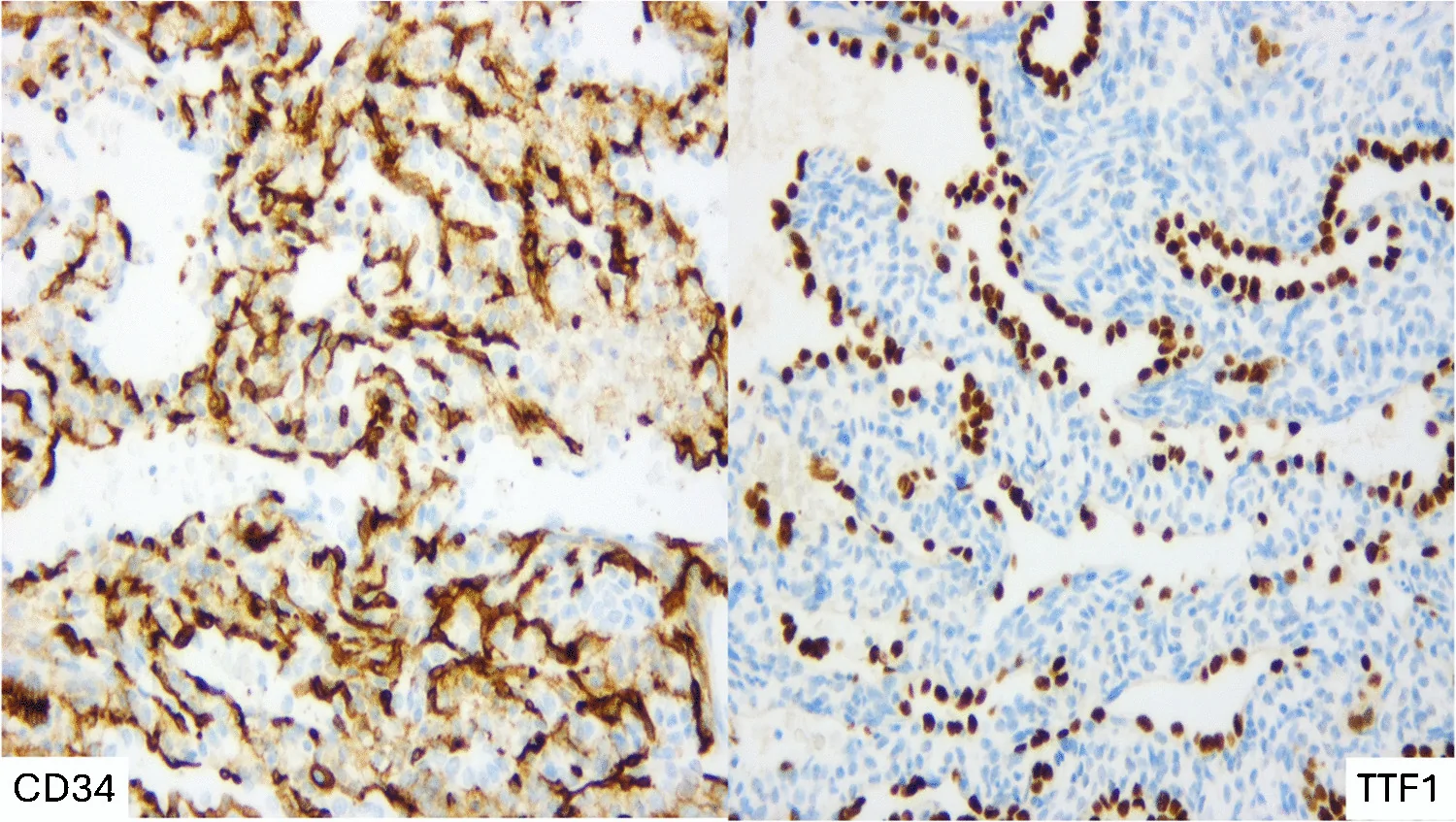
CD34 and TTF-1 immunohistochemistry. ×20 immunohistochemistry stains (*brown color*) showed significant CD34 staining (**a**) of lesional cells and TTF-1 (**b**) highlighted the pulmonary cuboidal epithelial cells lining the cavitary spaces

Definitive diagnosis relies on histopathologic evaluation to distinguish fetal lung interstitial tumor (FLIT) from congenital lung malformations and cystic lesions that can mimic CPAM on prenatal imaging [2, 4, 5, 8]. FLIT is characterized by a well-circumscribed fetal lung–like architecture with microcystic/airspace-like spaces lined by simple cuboidal epithelium and septal/interstitial expansion by bland immature mesenchymal cells, recapitulating canalicular-stage fetal lung [4, 8, 13]. This pattern helps distinguish FLIT from pleuropulmonary blastoma (PPB), which shows malignant primitive mesenchyme within the cyst wall (often subepithelial/cambium-like), with increased atypia/mitotic activity and sometimes rhabdomyoblastic differentiation, and is frequently associated with germline DICER1 alterations [15, 16]. In contrast, CPAM represents a developmental malformation without a true interstitial neoplastic proliferation [7]. In our case, immunohistochemistry showed TTF-1 highlighting the pulmonary epithelium lining the air spaces, associated with heterogeneous CD34 (Fig. 5), desmin, and SMA positivity and patchy/heterogeneous Ki-67 labeling; ALK, myogenin, and S100 were negative. β-Catenin demonstrated focal scattered nuclear positivity, a nonspecific finding reported in FLIT [4]. From a histopathologic standpoint, pulmonary interstitial glycogenosis (PIG) was also considered because of the patient’s neonatal age and the presence of clear cytoplasm within the interstitial cells. However, the morphologic features were not characteristic of PIG, and PAS-D staining did not demonstrate glycogen-containing cytoplasmic inclusions [7, 15–18].

FLIT is a rare entity, and prenatal detection is even more uncommon, with only three reported cases identified antenatally to date [9, 10, 14]. Most cases are diagnosed postnatally following the onset of symptoms, typically within the first 3 months of life. In reported series, the majority of affected infants were born at term and most commonly presented with respiratory distress, although clinical manifestations vary depending on lesion size and mass effect on adjacent structures [14].

Among prenatally detected cases, FLIT is most often identified in the third trimester. Phillips et al. reported the only case initially detected in the second trimester, which is also the first described occurrence in twins, with only one twin affected and presenting with significant respiratory distress at birth. In that case, antenatal corticosteroids were administered due to a presumed diagnosis of CPAM. Notably, Phillips et al. reported no interval growth of the lesion, which is atypical given the usual growth pattern observed in FLIT [9].

All other reported prenatal FLIT cases, including the present case, were first detected at approximately 33 weeks’ gestation, with no abnormal ultrasonographic findings earlier in pregnancy as seen in Table 1. On US, FLIT demonstrates hyperechogenicity compared to normal lung and typically well-circumscribed as described in Wang et al. and Phillips et al. [5, 9, 13, 14]. Third-trimester detection was frequently associated with additional prenatal findings, most notably fetal hydrops, as well as mediastinal shift, abnormal cardiac positioning, pleural effusion, and ascites. The development of fetal hydrops is likely secondary to the compressive effects of a rapidly enlarging mass on the heart and great vessels, leading to congestive heart failure and subsequent fluid accumulation in the lungs, lymphatic system, and body cavities, including pleural effusions, ascites, and polyhydramnios. The absence of rapid growth in the case reported by Phillips et al. may explain the lack of associated prenatal complications [9].

**Table 1:** Summary of FLIT detected prenatally

Case	Timing of first detection (GA)	Ultrasound (US) findings	MRI findings	Associated prenatal findings	Postnatal course	Survival	Pathology
Lazar et al., 2011 [13]	36 weeks	Solid hyperechoic mass	Solid hyperintense T2 mass	Mediastinal shift, eversion of the diaphragm, pleural effusion, and ascites	EXIT procedure at 37 weeks followed by immediate resection	Yes	Alveolar-like pattern of microcystic spaces and an expanded interstitium with immature-appearing mesenchymal cells
Phillips et al., 2020 [9]	26 weeks	Minimally hyperechoic solid 3.4×5.0×5.9 cm mass with mass effect and no cysts and vascularization	Slightly hyperintense on T2 with radiating bands; mediastinal shift and diaphragmatic flattening	No hydrops or polyhydramnios; mediastinal shift	Respiratory distress at birth; lobectomy on day 20; full recovery at 1 month	Yes	Spongy, hamartoma-like lesion with varying cystic/air spaces and increased interstitial thickness; interstitium contained uniform mesenchyme cells with ovoid nuclei and clear cytoplasm
Waelti et al., 2017 – case 2 [10]	33 weeks	At birth, US shows a 9.5-cm solid right upper lobe mass with no cysts, calcifications, vascularization, or infiltration of nearby structures	Not performed	Fetal hydrops, macrosomia, polyhydramnios, ascites; mediastinal compression with impaired venous return	Severe respiratory distress at birth; lobectomy	Yes	Solid, spongy, well-circumscribed lesion
Wang et al., 2023 [14]	33w2d	Prenatal US shows a solid, hyperechoic mass with cystic components; initially misdiagnosed as CPAM	Not performed	Mediastinal shift; progressive respiratory symptoms	Tachypnea, decreased breath sounds, and feeding difficulties at birth; lobectomy at 14 days; full recovery by 3 months	Yes	Spongy, well-circumscribed mass with cystic spaces
Hoang et al. (current case)	33w6d	Not seen on second-trimester US; identified ~3 weeks prior to MRI	Large heterogeneous T2-hyperintense mass with mediastinal shift, pleural effusion, ascites, and body wall edema	Fetal hydrops, ascites, pleural effusion, extreme cardiac levoposition	Surgery postnatally; no recurrence at 6-month follow-up	Yes	Expanding lung interstitium with lesional airspaces; interstitial cells contained clear cytoplasm and ovoid nuclei

*IVC*, inferior vena cava

All five prenatally detected cases were born with significant respiratory distress requiring surgical lobectomy for definitive treatment. Complete resection resulted in full recovery, with no reported recurrences [9, 10, 13, 14].

In summary, FLIT remains a rare congenital lung lesion with extremely limited reports of prenatal detection. This case adds to the growing but still sparse literature by highlighting the characteristic third-trimester presentation, rapid interval growth, and association with fetal hydrops that distinguish FLIT from more common congenital lung malformations. Given its significant radiographic overlap with entities such as CPAM and pleuropulmonary blastoma, FLIT should be considered in the differential diagnosis of late-presenting fetal lung masses, particularly when rapid growth and hydrops are present. Definitive diagnosis relies on histopathologic evaluation, as imaging alone cannot reliably differentiate benign from malignant lesions. Early recognition, multidisciplinary prenatal planning, and timely surgical intervention are essential for optimizing neonatal outcomes, as complete resection remains curative with excellent prognosis.

## Conclusion

FLIT should be considered in the differential diagnosis of late-presenting, rapidly enlarging fetal lung masses, particularly when associated with mediastinal shift or fetal hydrops. Although prenatal imaging is essential for detection and assessment, definitive diagnosis requires histopathologic confirmation, and complete surgical resection is curative.

## Data Availability

No datasets were generated or analysed during the current study.
